# Depression and Anxiety in Pediatric Patients with Beckwith–Wiedemann Syndrome: A Pilot Study

**DOI:** 10.3390/children11030342

**Published:** 2024-03-13

**Authors:** Grazia D’Onofrio, Annalisa Mastromatteo, Andrea Di Francesco, Antonio Izzi, Vincenzo Marchello, Aldo Manuali, Andreaserena Recchia, Maria Pia Tonti, Maria Lazzarina Russo, Maria Pia Affatato, Alessandra Rossato, Cecilia Giuntoli, Nicola Palladino, Michele Germano, Maria Rosa Pastore, Lazzaro Cassano

**Affiliations:** 1 Clinical Psychology Service, Health Department, Fondazione IRCCS Casa Sollievo della Sofferenza, San Giovanni Rotondo, 71013 Foggia, Italy; 2 Complex Unit of Maxillofacial Surgery and Otolaryngology, Fondazione IRCCS Casa Sollievo della Sofferenza, San Giovanni Rotondo, 71013 Foggia, Italy; a.mastromatteo@operapadrepio.it (A.M.); a.rossato@operapadrepio.it (A.R.); c.giuntoli@operapadrepio.it (C.G.); n.palladino@operapadrepio.it (N.P.); l.cassano@operapadrepio.it (L.C.); 3 Unit of Pediatric Maxillofacial Surgery, ASST Lariana, San Fermo della Battaglia, 22100 Como, Italy; andrea.difrancesco@asst-lariana.it; 4 Complex Unit of Anesthesia and Resuscitation II, Fondazione IRCCS Casa Sollievo della Sofferenza, San Giovanni Rotondo, 71013 Foggia, Italy; a.izzi@operapadrepio.it (A.I.); vincenzomarchello@libero.it (V.M.); a.manuali@operapadrepio.it (A.M.); a.recchia@operapadrepio.it (A.R.); mariapiatonti@alice.it (M.P.T.); 5 Complex Unit of Pediatrics, Fondazione IRCCS Casa Sollievo della Sofferenza, San Giovanni Rotondo, 71013 Foggia, Italy; maria.russo@operapadrepio.it (M.L.R.); m.pastore@operapadrepio.it (M.R.P.); 6 Unit of Odontostomatology, Fondazione IRCCS Casa Sollievo della Sofferenza, San Giovanni Rotondo, 71013 Foggia, Italy; m.affatato@operapadrepio.it; 7 Child Neuropsychiatry Service, Complex Unit of Pediatrics, Fondazione IRCCS Casa Sollievo della Sofferenza, San Giovanni Rotondo, 71013 Foggia, Italy; m.germano@operapadrepio.it

**Keywords:** Beckwith–Wiedemann syndrome, depression, anxiety, social aspects, family burden

## Abstract

The study’s aim was to determine the prevalence of depression and anxiety in children with Beckwith–Wiedemann syndrome (BWS) and their effects on social relationships and family acceptance. The Pediatric Symptom Checklist—35 items (PSC-35), Screen for Child Anxiety Related Emotional Disorders (SCARED), and the Vineland Adaptive Behavior Scale Second Edition (VABS-II) were administered to the children. The parental Acceptance Rejection/Control Questionnaire (PARQ/Control) and Zarit Burden Inventory (ZBI) were administered to parents. In total, 6 patients and 10 parents were included. Patients showed a significant presence of internalizing behavior in PSC-35 (mean, 7.66 ± 3.67), anxiety symptoms (SCARED: mean, 46.33 ± 17.50) and socialization difficulties (mean, 90.83 ± 10.09). Parents reported a perceived good acceptance (mean, 56.33 ± 1.03) and a moderate control (mean, 24.17 ± 1.83), but the burden level was ranked moderate to severe (mean, 59.33 ± 16.78). It was found that the severity of the burden level reported by parents was related to internalizing behavior (OR = 2.000; 95% CI = 0.479–3.521; *p* = 0.022) and anxiety symptoms (SCARED total score: OR = 3.000; 95% CI = 1.479–4.521; *p* = 0.005) of children. During psychological counseling in the context of BWS treatment, it is important to identify specific resources that can support patients and families in dealing with stress and identify any critical areas that could hinder the adaptation process.

## 1. Introduction

Beckwith–Wiedemann syndrome (BWS) is an epigenetic condition, a growth disorder that leads to an increase in body and organ size along with other prominent symptoms. The disease can be characterized by excess growth, enlargement of the tongue and abdominal wall defects (such as umbilical hernia or omphalocele, the protrusion of the intestine outside the navel). The frequency is one case every 13,700 births [1]. In 85–90% of cases, the syndrome is sporadic without family history [1]. BWS is determined by alterations in a particular group of imprinted genes located on chromosome 11p15.5 [2].

From a clinical point of view, we have various manifestations such as macroglossia, pathological enlargement of organs and body parts [3,4].

The most important event is the greater incidence of tumors in these children: in 10% of cases these children will develop malignant tumors such as Wilms’ tumor (a rare renal tumor) or hepatoblastoma (a tumor of liver origin), but also non-malignant tumors [4,5]. Particular manifestations can be wrinkles in the earlobes or abnormalities in the auricle, large and prominent eyes and other anomalies in the head such as the presence of a nevus flammeus, or a flat angioma usually at the frontal level visible as a red spot that may disappear with growth [3,4].

The diagnosis is based on clinical criteria and confirmed by molecular or cytogenetic testing [1,6].

There is no specific therapy. Some manifestations (such as omphalocele, macroglossia or skeletal asymmetry) are treated surgically [6,7]. Furthermore, regarding tumor surveillance, children with BWS are offered abdominal ultrasound scans every 3 months until they are seven years of age [1,8].

Therefore, relevant social-psychological consequences result, such as anxiety and depression [9]. In particular, difficulty speaking due to macroglossia provokes isolation and reduced social interactions [10].

This psychological situation is even more difficult in those conditions in which an increase in oncological risk is not associated with the possibility of carrying out specific monitoring. These reflections require specific attention to this emotional corpus which undoubtedly conditions the emotional well-being, not only of the child, but also of the entire family unit.

In light of these assumptions, the aim of the present study was to determine the prevalence of depression and anxiety in children with BWS and their effects on social relationships and family acceptance.

## 2. Materials and Methods

This study was conducted according to the Declaration of Helsinki, the Guidelines for Good Clinical Practice and the Guidelines for Strengthening the Reporting of Observational Studies in Epidemiology [11].

### 2.1. Study Sample

From December 2021 to December 2023, pediatric patients with BWS who had consecutively attended the Multidisciplinary Assessment Unit for Pediatric Malformative Surgery of the Complex Unit of Maxillofacial Surgery and Otolaryngology of the Institute of Hospitalization and Scientific Care (IRCCS) “Casa Sollievo della Sofferenza” were selected for possible enrollment in the study. We obtained written informed consent for the research from each relative or legal guardian. All subjects were Caucasians, with the exclusion of people of Jewish, Eastern European, or North African descent. Most of the people had southern Italian origins, and were from families who had lived in southern Italy for at least three generations.

Inclusion criteria were: (1) age ≥ 8 years; (2) diagnosis of BWS according to the consensus guideline [1] criteria; (3) craniofacial surgeon assessment. The exclusion criteria were: (1) absence of documentation about clinical examinations; (2) patients younger than 8 years of age at the time of most recent clinical examination; (3) presence of neurological and psychiatric disorders (e.g., autism spectrum disorder, epilepsy).

### 2.2. Demographic and Clinical Assessment

In all patients, the following data were collected through a specific information sheet:

1. Demographic indices: age, gender;
2. Anthropometric parameters (height, weight) and medical history.

For the parents, age and level of education were collected.

### 2.3. Emotional and Behavioral Assessment

In order to recognize cognitive, emotional, and behavioral problems, the Pediatric Symptom Checklist—35 items (PSC-35)—was administered. It is a psychosocial screening instrument directed to parents and rated as “Never”, “Sometimes”, or “Often” present and scored 0, 1, and 2, respectively. The total score is calculated by adding together the score for each of the 35 items. For children and adolescents aged 6 through 16, a cutoff score of 28 or higher indicates psychological impairment [12,13].

For anxiety symptoms, the Screen for Child Anxiety Related Emotional Disorders (SCARED) was administered [14,15,16,17,18]. It is a self-report screening questionnaire for youth, 8–18 years old and their parents to complete in about 10 min. The SCARED screening questionnaire was developed as an instrument for both children and their parents that would encompass several DSM-5 categorizations of anxiety disorders: somatic/panic, generalized anxiety, separation anxiety, social phobia, and school phobia. Each question measures the frequency or intensity of symptoms or behaviors. In particular, a total score of ≥25 may indicate the presence of an anxiety disorder. According to the five subscales, a score of 7 may indicate panic disorder or Significant Somatic Symptoms (PD/SSS), a score of 9 may indicate Generalized Anxiety Disorder (GAD), a score of 5 may indicate Separation Anxiety Disorder (SepAD), a score of 8 may indicate Social Anxiety Disorder (SocAD), and a score of 3 may indicate Significant School Avoidance (SSA). This assessment has been found to be both valid and reliable in research settings [19].

### 2.4. Psycho-Motor, Functional and Social Evaluation

The adaptive level of functioning was assessed by Vineland Adaptive Behavior Scale Second Edition (VABS-II) [19], a standardized interview administered to the parents about the capabilities of children in dealing with everyday life (i.e., Communication, Daily Living Skills, Socialization and Motor Skills). Individual items are rated on a scale of 0 (never performs skill independently) to 2 (often performs skill independently). Item scores within a subdomain are totaled and raw scores are transformed to v-Scale Scores (mean = 15, SD = 3). V-Scale scores of subdomains are then totaled and transformed to Domain scores (mean = 100, SD = 15). Domain scores are totaled and transformed to an Adaptive Behavior Composite (mean = 100, SD = 15).

### 2.5. Parental Acceptance and Burden Assessment

The Parental Acceptance-Rejection/Control Questionnaire (PARQ/Control) for mothers and fathers is an inventory of 60 items (for parental acceptance/rejection) and 13 items (for parental control) on a Likert scale (4 = almost always true to 1 = almost never true), where parents record their acceptance/rejection and control behaviors towards their children [20]. Regarding parental acceptance/rejection, the questionnaire elicits information about perceived warmth/affection (or coldness, lack of warmth), hostility/aggression, indifference/neglect, and undifferentiated rejection; the total score ranges from a low of 60 (maximum perceived acceptance) through a high of 240 (maximum perceived rejection). Regarding parent control, the questionnaire elicits information about permissive/strictness behaviors using the following score ranges: 8–16 (low-permissive control), 17–24 (moderate control), 25–28 (tight control), and 29–32 (strict/restrictive control).

The burden level of the parents was assessed by Zarit Burden Inventory (ZBI) [21], a self-report containing 22 items. The response method includes a Likert scale from 0 (never) to 4 (almost always), based on the degree of agreement with the single item. The total sum of the scores for the individual items is between 0 (no care burden) and 88 (maximum level of care burden). Values below 20 indicate little or no care burden, values of 21–40 indicate mild to moderate burden, values of 41–60 indicate moderate to severe burden, and values of 61–88 indicate severe care load. A score higher than 24–26 identifies those caregivers for whom further investigation and possible support intervention would be indicated.

### 2.6. Statistical Analysis

Demographic and clinical characteristics of children with BWS were reported as averages and standard deviations or frequencies and percentages depending on whether they were continuous variables or categorical variables. For continuous variables, normal distribution was verified by the Shapiro–Wilk normality test and the one-sample Kolmogorov–Smirnov test. Associations between patient internalizing behavior and anxiety presence/absence and burden level of parents were reported as odds ratios (OR) along with their 95% confidence interval (CI).

All the statistical analyses were made with the R Ver. 2.8.1 statistical software package (The R Project for Statistical Computing; available at URL http://www.r-project.org/ (accessed on 2 January 2024)). Tests in which the *p*-value was smaller than the type I error rate α = 0.05 were declared significant.

## 3. Results

### 3.1. Clinical Characteristics of Patients

In the course of the enrolment period, 6 children were screened for inclusion in the study, one male and five females, with a mean age of 12.20 ± 4.01 years. As indicated in Table 1, in all patients the average weight-to-height ratio was normal. Only one child was born prematurely with complications at birth. All patients underwent one or more surgeries (mean, 1.67 ± 0.81) of glossectomy. In addition, two children underwent another surgery (respectively, epiphysiodesis and omphalocele).

### 3.2. Emotional, Behavioral and Adaptive Aspects

As shown in Table 2, in terms of cognitive, emotional, and behavioral aspects, the patients showed a significant presence of internalizing behavior (mean, 7.66 ± 3.67), although the PSC-35 total score did not indicate a clinical risk.

The patients reported a significant presence of anxiety symptoms (SCARED total score: mean, 46.33 ± 17.50). In particular, they showed a high score for PD/SSS (mean, 9.50 ± 6.09), GAD (mean, 12.50 ± 5.65), SepAD (mean, 6.50 ± 2.59), SocAD (mean, 13.50 ± 6.28), and SSA (mean, 4.17 ± 2.79).

The adaptive behavior (Table 3) was slightly below the average (95.04 ± 3.39). In particular, the socialization aspects were more compromised (mean, 90.83 ± 10.09).

### 3.3. Burden, Acceptance/Rejection and Control Behaviors of the Parents towards Their Children

As shown in Table 4, ten parents were interviewed (four males and six females), with a mean age of 45.10 ± 5.04 years. There was a perceived good acceptance (mean, 56.33 ± 1.03) and a moderate control (mean, 24.17 ± 1.83). However, the burden level was ranked as moderate to severe (mean, 59.33 ± 16.78).

In Table 5, it is shown that the burden level severity of the parents was related to internalizing behavior (OR = 2.000; 95% CI = 0.479–3.521; *p* = 0.022) and anxiety symptoms (SCARED total score: OR = 3.000; 95% CI = 1.479–4.521; *p* = 0.005) of the children. In particular, PD/SSS (OR = 3.243; 95% CI = 2.563-3.922; *p* < 0.0001), GAD (OR = 2.696; 95% CI = 0.900–4.492; *p* = 0.015), SepAD (OR = 2.403; 95% CI = 0.376–4.430; *p* = 0.030), SocAD (OR = 2.056; 95% CI = 0.412–3.699; *p* = 0.026), and SSA (OR = 2.876; 95% CI = 1.692–4.059; *p* = 0.003) of the children were related to increase of the burden level of the parents.

## 4. Discussion

In the present pilot study, we characterized the psycho-behavioral aspects in six unrelated BWS pediatric patients attending the Multidisciplinary Assessment Unit for Pediatric Malformative Surgery of IRCCS Casa Sollievo della Sofferenza.

Our results indicated the significant presence of internalizing behavior (depression and anxiety) and socialization problems in children with BWS. Currently, there is no scientific evidence directly linking BWS to depression and anxiety. BWS is mainly characterized by excess growth and development of some organs and tissues, such as the tongue, internal organs and extremities [1]. This condition can pose physical and emotional challenges for patients and their families [9,22]. Confirming this, our results reported a significant level of parental burden. Moreover, in our pilot study, a relation between depression and anxiety of the children with BWS and burden level severity of parents was observed.

In cases of diseases with onset in childhood, what must be accepted is not the loss of previously acquired functions and quality of life, but the difficulty in going through the phases of the family life cycle that each person would expect in normal conditions. For parents, it may mean not being able to play with their children, wondering if they will be able to attend school, if the parental couple will be able to recover autonomy and marital intimacy, or if the care commitment will saturate every family space. For the child, it may mean growing up by combining the effort to keep himself projected on his future, trying to lead a life as “normal” as possible, with the anguished feeling of a fragile body and the fear of not being able to grow up [23].

Living with a disease, such as BWS, with a cancer onset risk, has important psychological repercussions, as it has implications for every aspect of life and on numerous choices and decisions, from school, to work, to free time, to personal relationships [24].

The diagnosis of BWS overwhelms not only the lives of those affected, but also those of their family. The family is called to face multiple challenges; the load of tension and suffering can sometimes be the driving force for greater closeness and mutual support, but in other cases cause conflicts and fractures.

The emotional reaction to the diagnosis usually goes through several phases [25]: uncertainty and confusion; bewilderment, anguish, loneliness, opposition and isolation, with the appearance of defense mechanisms such as denial of the illness; anger, which also represents a defense against the feeling of helplessness; sadness, with withdrawal into oneself and internal processing of what happens; adaptation, that is, finally being able to face the disease by accepting its limitations but investing in everything that is still possible.

BWS poses clinical difficulties. For this disease there are no large case studies to draw on, there may be no specific therapies and diagnostic tests and even just getting the diagnosis is a tortuous and long journey [22]. There is almost never an effective cure but the possible interventions address the symptoms to improve the quality of life [22].

One of the most frequent psychological aspects in people with BWS is the perception of being poorly understood and supported by society. Many experience insecurity and poor access to care and assistance compared to other types of patients [26]. The lack of social recognition leads to an experience of isolation and helplessness; having continually to explain the illness to others leads to frustration, anger and often social withdrawal [26].

Living with an illness can bring about significant changes. It may be necessary to move house, both due to mobility problems and architectural barriers, and for greater proximity to family members who can provide assistance. Many caregivers are forced to reduce or abandon work: among parents of children with diseases, very often one of the two, usually the mother, has to stop working in order to care for the child.

Children affected by BWS and their families therefore particularly need psychological support that helps them mobilize resources for continuous readjustment to the challenges posed by the pathology [22,27]. A fundamental role is played by voluntary associations [27]. The associations play an essential role because not only do they provide information, but generally the volunteers are the same people who have the disease or are caregivers who have experienced everything first hand and with whom it is possible to discuss. For the newly diagnosed, there is the possibility of finding a model and positive reinforcement in the experience of others, and for the volunteers, who are themselves ill. There is a greater sense of self-efficacy and personal fulfilment thanks to the perception of having given help concrete to others.

The study has limitations especially due to the number of the sample examined and the regression analysis, but it indicates a way to delve deeper into aspects linked to emotional regulation, depression/withdrawal, highlighting a connection with the distinctive relational methods of parents. The next step of our study will be to implement sample case studies and detect any psycho-behavioral changes in the patients and in the caregiver’s stress after having performed a course of psychotherapy.

## 5. Conclusions

With these premises, psychological counseling should constitute an integral part of the treatment path aimed at children with BWS, especially in highly complex cases such as BWS, and the central question is “what to evaluate?”. Borrowing the term resilience from physics, we mean the ability to face adverse events through evolutionary change. The challenge, essentially, is not to maintain one’s impassivity in the face of the event, but to change together with it by recognizing the pain and without losing the ability to engage in life in an active and proactive way [28]. During psychological counseling in the context of the treatment of diseases, such as BWS, it is important to identify the specific resources that can support patients and families in dealing with stress and identify any critical areas that could hinder the adaptation process, in order to be able to address them.

## Figures and Tables

**Table 1:** Demographic and clinical characteristics of the patients.

	Patients (*n* = 6)
**Sex** (Male/Female)—*n* (%)	1 (16.66%)/5 (83.33%)
**Age**—(mean ± sd)	12.20 ± 4.01
**Weight**—(mean ± sd)	50.33 ± 8.96
**Height**—(mean ± sd)	152.83 ± 13.29
**Premature birth**—*n* (%)	1 (16.66%)
**Complications at birth**—*n* (%)	1 (16.66%)
**N of previous surgeries**—(mean ± sd)	1.67 ± 0.81
**Surgery area**	
Glossectomy—*n* (%)	6 (100%)
Epiphysiodesis—*n* (%)	1 (16.66%)
Omphalocele—*n* (%)	1 (16.66%)

**Table 2:** Emotional and behavioral aspects of the patients assessed by Pediatric Symptom Checklist—35 items (PSC-35), and Screen for Child Anxiety Related Emotional Disorders (SCARED).

	Patients (*n* = 6)	Cut Off
**PSC-35**		
Attention disease—(mean ± sd)	4.67 ± 1.50	<7
Internalizing behavior—(mean ± sd)	7.66 ± 3.67	<5
Externalizing behavior—(mean ± sd)	4.67 ± 2.65	<7
Total Score—(mean ± sd)	26.67 ± 5.82	<28
**SCARED**		
PD/SSS—(mean ± sd)	9.50 ± 6.09	7
GAD—(mean ± sd)	12.50 ± 5.65	9
SepAD—(mean ± sd)	6.50 ± 2.59	5
SocAD—(mean ± sd)	13.50 ± 6.28	8
SSA—(mean ± sd)	4.17 ± 2.79	3
Total Score—(mean ± sd)	46.33 ± 17.50	25

Legend: PD/SSS, Panic Disorder or Significant Somatic Symptoms; GAD, Generalized Anxiety Disorder; SepAD, Separation Anxiety Disorder; SocAD, Social Anxiety Disorder; SSA, Significant School Avoidance.

**Table 3:** Psycho-motor, Functional and Social aspects of the patients assessed by Vineland Adaptive Behavior Scale Second Edition (VABS-II).

	Patients (*n* = 6)
Communication—(mean ± sd)	93.50 ± 7.31
Daily Living Skills—(mean ± sd)	95.83 ± 5.84
Socialization—(mean ± sd)	90.83 ± 10.09
Motor Skills Domain—(mean ± sd)	100.00 ± 0
Adaptive Behavior Composite—(mean ± sd)	95.04 ± 3.39

**Table 4:** Burden, acceptance/rejection and control behaviors of the parents towards their children, assessed by Zarit Burden Inventory (ZBI) and Parental Acceptance-Rejection/Control Questionnaire (PARQ/Control).

	Parents (*n* = 10)
**Sex** (Male/Female)—*n* (%)	4 (40.00%)/6 (60.00%)
**Age**—(mean ± sd)	45.10 ± 5.04
**PARQ/Control**	
Acceptance/rejection—(mean ± sd)	56.33 ± 1.03
Control—(mean ± sd)	24.17 ± 1.83
**ZBI**—(mean ± sd)	59.33 ± 16.78

**Table 5:** Internalizing behavior (measured by PSC-35) and anxiety symptoms (measured by SCARED) of the children according to the burden level of parents.

	*p* Value	OR	95% CI
**Internalizing behavior**	0.022	2.000	0.479–3.521
**SCARED Total score**	0.005	3.000	1.479–4.521
PD/SSS	<0.0001	3.243	2.563–3.922
GAD	0.015	2.696	0.900–4.492
SepAD	0.030	2.403	0.376–4.430
SocAD	0.026	2.056	0.412–3.699
SSA	0.003	2.876	1.692–4.059

## Data Availability

The data presented in this study are available on request from the corresponding author. The data is not publicly available due to patient privacy concerns.
